# 超声支气管镜引导下的经支气管针吸活检对肺癌的诊断价值

**DOI:** 10.3779/j.issn.1009-3419.2010.05.10

**Published:** 2010-05-20

**Authors:** 加源 孙, 宝惠 韩, 俭 张, 珩 赵, 大江 戚, 洁 沈, 爱琴 顾

**Affiliations:** 200030 上海，上海交通大学附属胸科医院呼吸内科，卫生部呼吸内镜诊疗基地 Department of Respiratory Medicine, Shanghai Chest Hospital, Shanghai Jiaotong University, Shanghai 200030, China

**Keywords:** 超声支气管镜, 经支气管针吸活检, 肺肿瘤, 淋巴结, Endobronchial ultrasound, Transbronchial needle aspiration, Lung neoplasms, Lymph node

## Abstract

**背景与目的:**

本研究旨在评价超声支气管镜引导下的经支气管针吸活检（endobronchial ultrasoundguided transbronchial needle aspiration, EBUS-TBNA）对肺癌的诊断价值和安全性。

**方法:**

于2009年7月-2010年2月，对95例胸部CT检查显示纵隔/肺门淋巴结肿大和/或胸内气管或支气管旁肿块患者进行EBUS-TBNA，并未采取现场细胞学方法进行检测。

**结果:**

95例患者中，其中经病理学检查和临床随访验证新发肺癌患者60例。60例肺癌患者共穿刺112组淋巴结，肺内肿块11例。60例肺癌患者中通过EBUS-TBNA明确诊断58例，假阴性2例，敏感性为96.67%、特异性100%。EBUS-TBNA过程安全，全部病例无并发症发生。

**结论:**

EBUS-TBNA是诊断肺癌安全、有效的方法。

## Abstract (en)

**Background and objective:**

The aim of this study is to evaluate diagnostic yield and the safety of endobronchial ultrasound-guided transbronchial needle aspiration (EBUS-TBNA) in the diagnosis of bronchogenic carcinoma.

**Methods:**

Between July, 2009 and February, 2010, 95 patients with mediastinal/hilar lymphadenopathy and/or intrathoracic peritracheal or peribronchial masses previously detected with CT scan underwent EBUS-TBNA. No rapid onsite cytology was performed.

**Results:**

In all 95 patients, 60 cases were newly diagnosed lung cancer through the pathological examination and clinical follow-up certification. In 60 lung cancer cases, 112 samples were obtained from lymph nodes (LNs) and 11 samples were obtained from intrapulmonary lesions. Fifty-eight cases of patients were diagnosed, false negative in 2 cases. Sensitivity and specificity of EBUS-guided TBNA method in distinguishing benign from malignant LNs or thoracic masses were 96.67% and 100%, respectively. There was any major complication in this series, the procedure was uneventful.

**Conclusion:**

EBUSTBNA seemed a safe and effective technique in making bronchogenic carcinoma diagnosis for mediastinal/hilar LNs and intrapulmonary masses.

超声支气管镜引导下的经支气管针吸活检（endobronchial ultrasound-guided transbronchial needle aspiration, EBUS-TBNA）是2002年开始研发的新技术^[1]^，2007年即已被美国国家综合癌症网络（National Comprehensive Cancer Network, NCCN）和美国胸科医师学会（American College of Chest Physicians, ACCP）肺癌指南推荐为肺癌术前评估的重要工具^[2, 3]^。EBUS-TBNA的主要适应症是：①肺癌患者淋巴结（lymph node, LN）分期；②诊断肺内肿瘤；③诊断不明原因的肺门和/或纵隔LN肿大；④诊断纵隔肿瘤^[1]^。EBUS-TBNA在2008年引入中国并投入临床使用，我院于2009年6月引进该项设备和技术，研究者在完成5例学习曲线训练后，于2009年7月10日-2010年2月24日共行EBUSTBNA检查95例，其中经病理学检查和临床随访验证新发肺癌患者60例。我们对EBUS-TBNA在肺癌原发灶及LN转移诊断的作用和安全性进行了初步研究，报道如下。

## 1 材料与方法

### 1.1 一般资料

本组患者60例，男46例，女14例；年龄36岁-84岁，平均60.03岁。其中门诊患者18例，住院患者42例。病例选择：患者胸部CT检查发现纵隔/肺门LN肿大（≥1 cm）和/或位于气管或支气管周围的胸内肿块。患者同意进行EBUS-TBNA检查，无相关禁忌症。

### 1.2 EBUS-TBNA操作

手术前至少6 h禁食禁水，操作前建立静脉通路，杜冷丁25 mg-50 mg肌注，力月西2 mg-5 mg静脉推注，2%利多卡因经口滴注，加用7%利多卡因3喷-5喷。先经口行普通支气管镜检查，然后使用搭载电子凸阵扫描的超声支气管镜（BF-UC260F-OL8, Olympus Ltd, Tokyo, Japan）检查目标LN和周围血管，LN检查根据国际分期系统^[4]^。超声顶端安放水囊，扫描频率7.5 MHz，超声影像的加工通过超声图像处理装置（EU-C60, Olympus Ltd），冻结超声图像的情况下记录目标LN直径，使用22号穿刺吸引针（NA-201SX-4022, Olympus Ltd）在实时超声指导下进行穿刺吸引，确认穿刺针进入靶区后，来回移动穿刺针进行抽吸。穿刺之前启用多普勒功能排除穿刺针穿入血管。推荐目标LN和肿块进行3次穿刺。如能拿到组织标本，2次穿刺可满足需要，并未采取现场细胞学方法进行检测。细胞学涂片检查由同一位有经验的病理医生盲法完成，得到的组织学标本固定在福尔马林液中，石蜡包埋制成切片后行组织学检查。

### 1.3 TBNA结果判断

TBNA涂片中如果可见多个淋巴细胞团，认为TBNA穿刺到LN；如未见到淋巴细胞，认为穿刺到肿块；如果为大量红细胞或有核细胞很少，则认为TBNA穿刺失败。TBNA涂片中见到明确的恶性肿瘤细胞，即使不能区别类型或分化程度，均认为TBNA结果阳性；涂片中见到高度可疑的恶性肿瘤细胞时，如果患者临床表现高度怀疑肺癌，或其它组织学或细胞学检查证明为肺癌，则也认为TBNA结果阳性，否则判断为阴性；每例患者任何一个部位TBNA结果阳性，则认为TBNA结果总结果阳性；全部部位TBNA结果阴性，则认为TBNA总结果阴性。

### 1.4 统计分析

EBUS-TBNA的诊断结果通过开胸手术、纵隔镜、胸腔镜等其它病理学检查或临床随访验证，患者根据相应的检查结果进行治疗。根据标准定义计算敏感性、特异性。应用SPSS 11.5统计软件进行统计分析。

## 2 结果

### 2.1 临床诊断结果

60例肺癌患者中，非小细胞肺癌49例：低分化癌12例，鳞癌16例，腺癌21例；小细胞癌8例；低分化伴小细胞癌3例。

### 2.2 穿刺结果

60例患者共穿刺112组LN（2R组5例次，4R组28例次，4L组10例次，7组34例次，10L组5例次，10R组9例次，11L组4例次，11R组12例次，12R组4例次，12L组1例次），肺内肿块11例（右上肺肿块8例次，右下肺肿块3例次），每个部位共进行1次-4次穿刺，平均1.98次（表 1）。

**1:** 60例实时EBUS-TBNA患者穿刺纵隔/肺门LN和肺内肿块的位置和结果 Results of real-time EBUS-TBNA in 60 patients with mediastinal/hilar lymph nodes and intrapulmonary masses by location

LN station /intrapulmonary mass	Nodes/masses (*n*)	Cell smears positive (*n*)	Tissue specimens positive (*n*)	Total positive (*n*)	Nodes/masses diagnosed (%)
2R	5	5	4	5	100.00
4R	28	23	18	23	82.14
4L	10	7	8	8	80.00
7	34	27	14	28	82.35
10L	5	4	2	4	80.00
10R	9	7	5	7	77.78
11L	4	3	3	3	75.00
11R	12	7	6	7	58.33
12R	4	2	2	3	75.00
12L	1	1	1	1	100.00
Right upper lobe	8	7	5	8	100.00
Right lower lobe	3	1	1	2	66.67
Total	123	94	69	99	80.49
LN: lymph node.					

60例患者中54例（90%）通过TBNA获得组织学证据，其中42例具有明确诊断的意义。60例肺癌患者通过EBUS-TBNA共诊断58例（其中非小细胞肺癌48例：低分化12例，鳞癌16例，腺癌20例；小细胞癌7例，低分化合并小细胞癌3例（代表病例见图 1-图 4）。

**1:**
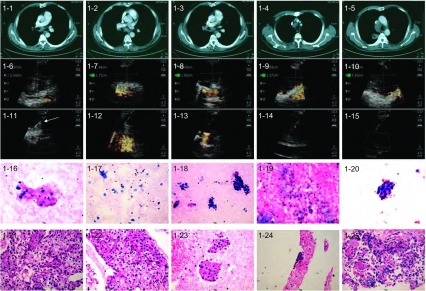
EBUS-TBNA诊断63岁患者右上叶低分化肺癌LN转移。1-1–1-5：胸部CT示LN增大（从左至右依次为7、10R、11R、2R、4R组LN）；1-6–1-10：EBUS定位和测量LN（从左至右依次为7、10R、11R、2R、4R组LN）；1-11–1-15：EBUS-TBNA目标LN（从左至右依次为7、10R、11R、2R、4R组LN），穿刺针（箭头所指方向）在穿刺部位内实时穿刺；1-16–1-20：目标LN TBNA细胞学结果证实为低分化癌（HE, ×20）（从左至右依次为7、10R、11R、2R、4R组LN）；1-20–1-25：目标LN TBNA组织学结果证实为低分化癌（HE, ×20）（从左至右依次为7、10R、11R、2R、4R组LN）。 Poorly differentiated lung cancer with LNs metastasis diagnosed by EBUS-TBNA in a 63-year-old patient with lung cancer of the right upper lobe. 1-1–1-5: Chest CT shows enlarged LNs (7, 10R, 11R, 2R, 4 R from left to right); 1-6–1-10: Localization and measurement of LNs (7, 10R, 11R, 2R, 4R from left to right) by EBUS; 1-11–1-15: EBUS-TBNA of LNs(7, 10R, 11R, 2R, 4R from left to right) with a needle (arrow) within the aspiration site; 1-16–1-20: TBNA cytological results of LNs(7, 10R, 11R, 2R, 4R from left to right) demonstrated poorly-differentiated cancer (HE, ×20); 1-20–1-25: TBNA tissue specimens of LNs(7, 10R, 11R, 2R, 4R from left to right) indicated poorly-differentiated cancer (HE, ×20).

**2:**
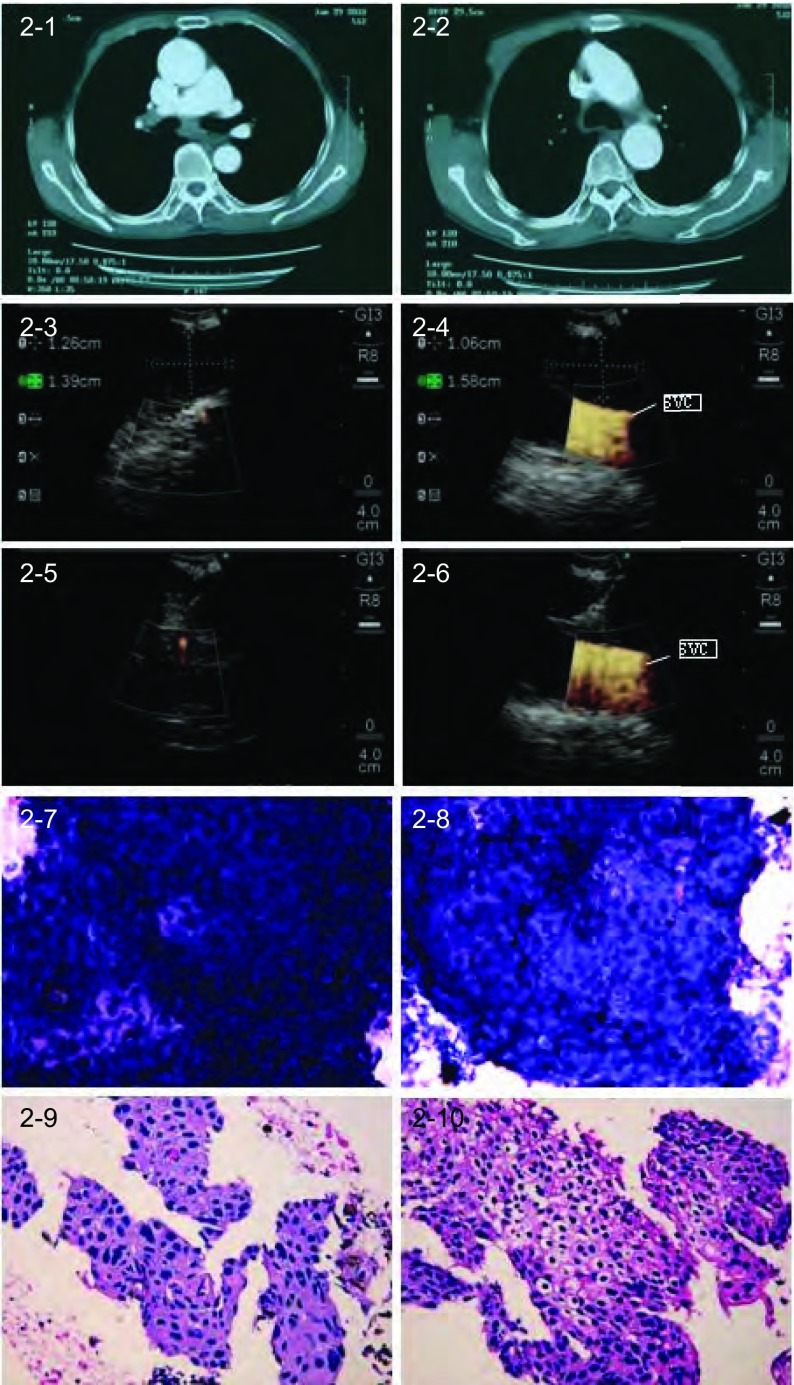
EBUS-TBNA诊断51岁患者右下叶鳞癌LN转移。2-1–2-2：胸部CT示LN增大（从左至右依次为7、4R组LN)；2-3–2-4：EBUS定位和测量LN（从左至右依次为7、4R组LN)；2-5–2-6：EBUS-TBNA目标LN（从左至右依次为7、4R组LN)，穿刺针在穿刺部位内实时穿刺；2-7–2-8：目标LN TBNA细胞学结果证实为鳞癌（HE, ×20）（从左至右依次为7、4R组LN）；2-9–2-10：目标LN TBNA组织学结果证实为鳞癌（HE, ×20）（从左至右依次为7、4R组LN）。SVC：上腔静脉。 Squamous cell lung carcinoma with LNs metastasis diagnosed by EBUS-TBNA in a 51-year-old patient with lung cancer of the right lower lobe. 2-1–2-2: Chest CT shows enlarged LNs (7, 4R from left to right); 2-3–2-4: Localization and measurement of LNs (7, 4R from left to right) by EBUS; 2-5–2-6: EBUS-TBNA of LNs (7, 4R from left to right) with a needle within the aspiration site; 2-7–2-8: TBNA cytological results of LNs (7, 4R from left to right) demonstrated squamous cell carcinoma (HE, ×20); 2-9–2-10: TBNA tissue specimens of LNs (7, 4R from left to right) indicated squamous cell carcinoma (HE, ×20). SVC : superior vena cava.

**3:**
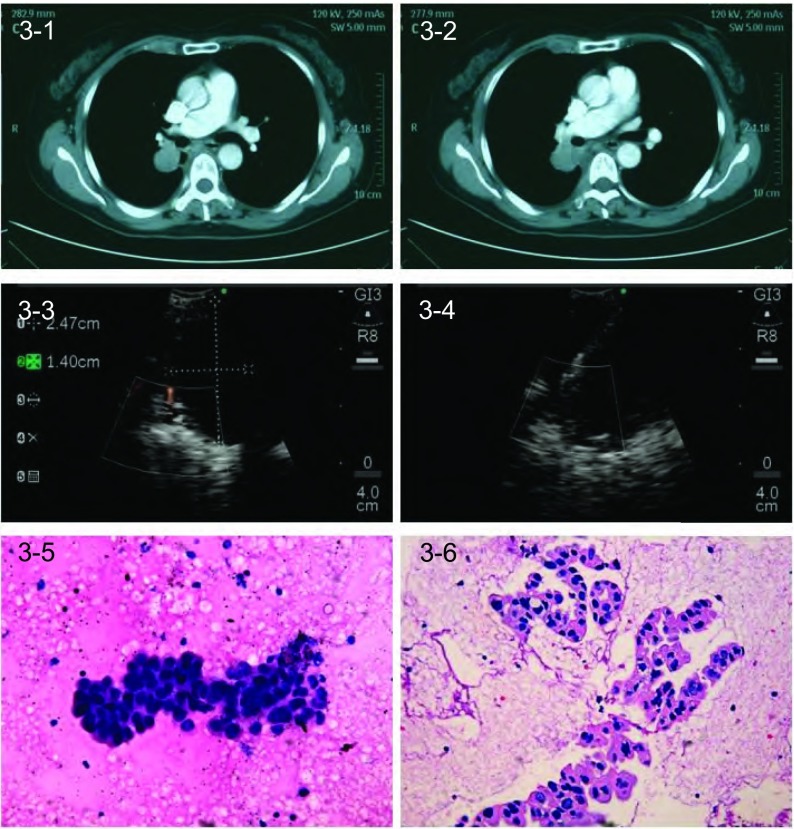
EBUS-TBNA诊断56岁患者右肺腺癌。3-1–3-2：胸部CT示右上肺肿块和11R LN融合；3-3：EBUS定位和测量右上肺肿块；3-4：EBUS-TBNA右上肺肿块，穿刺针在穿刺部位内实时穿刺；3-5：右上肺肿块TBNA细胞学结果证实为腺癌（HE, ×20）；3-6：右上肺肿块TBNA组织学结果证实为腺癌（HE, ×20）。 Lung adenocarcinoma diagnosed by EBUS-TBNA in a 56-yearold patient with lung cancer of the right lung. 3-1–3-2: Chest CT shows a right-upper lung mass coalesced with 11R LN; 3-3: Localization and measurement of right-upper lung mass by EBUS; 3-4: EBUS-TBNA of right-upper lung mass with a needle within the aspiration site; 3-5: TBNA cytologicl results of right upper lung mass demonsted adenocarcinoma (HE, ×20); 3-6: TBNA tissue specimens of right upper lung mass indicated adenocarcinoma (HE, ×20).

**4:**
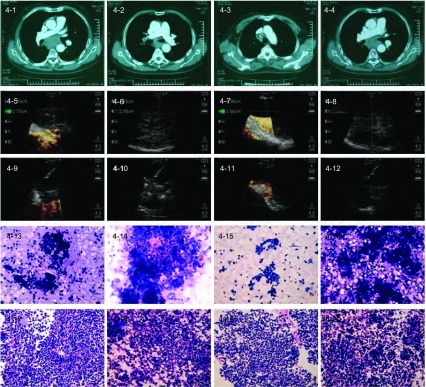
EBUS-TBNA诊断69岁患者纵隔型小细胞肺癌。4-1–4-4：胸部CT示LN增大（从左至右依次为10L、7、4R、10R组LN）；4-5–4-8：EBUS定位和测量LN（从左至右依次为10L、7、4R、10R组LN）；4-9–4-12：EBUS-TBNA LN（从左至右依次为10L、7、4R、10R组LN），穿刺针在穿刺部位内实时穿刺；4-13–4-16：目标LN TBNA细胞学结果证实为小细胞癌（HE, ×20）（从左至右依次为10L、7、4R、10R组LN）；4-16–4-20：目标LNTBNA组织学结果证实为小细胞癌（HE, ×20）（从左至右依次为10L、7、4R、10R组LN）。 Mediastinal small cell lung carcinoma with LNs involved diagnosed by EBUS-TBNA in a 69-year-old patient. 4-1–4-4: Chest CT shows enlarged LNs (10L, 7, 4R, 10R from left to right); 4-5–4-8: Localization and measurement of LNs (10L, 7, 4R, 10R from left to right) by EBUS; 4-9– 4-12: EBUS-TBNA of LNs (10L, 7, 4 R, 10R from left to right) with a needle within the aspiration site; 4-13–4-16: TBNA cytological results of LNs (10L, 7, 4R, 10R from left to right) demonstrated small cell lung carcinoma (HE, ×20); 4-16–4-20: TBNA tissue specimens of LNs (10L, 7, 4R, 10R from left to right) indicated small cell lung carcinoma (HE, ×20).

### 2.3 结果分析

在60例患者中，58例肺癌患者中通过EBUSTBNA明确诊断，假阴性2例，敏感性为96.67%，特异性为100%。

### 2.4 并发症观察

患者均能很好耐受操作，除外3例患者因术中咳嗽难以控制，未能充分进行检查。操作过程中除内窥镜观察到穿刺点少许出血外，未发现气胸、纵隔气肿、纵隔大血管破裂出血等严重并发症。

## 3 讨论

超声支气管镜（endobronchail ultrasound, EBUS）是一种在支气管镜前端安装超声探头的设备，结合专用的吸引活检针，可以在实时超声引导下行经支气管针吸活检术（transbronchial needle aspiration, TBNA），搭载的电子凸阵扫描的彩色能量多普勒同时可以帮助确认血管的位置，防止误穿血管。穿刺吸引针的外径为22 G，在部份病例可以获得组织样品，具有安全、准确的特点。

Yasufuku报道^[5]^应用EBUS-TBNA对70例患者纵隔和肺门LN进行穿刺，区分良恶性纵隔LN的敏感性、特异性、准确率分别为95.7%、100%和97.1%；Herth报道^[6]^502例纵隔或肺门LN肿大患者，经EBUS-TBNA穿刺572枚LN，诊断敏感性为94%、特异性为100%、阳性预测值为100%，无并发症出现；有研究^[7]^表明EBUS-TBNA诊断肺癌的平均敏感性为90%、假阴性率为20%。传统TBNA根据CT定位进行盲穿，结果变动较大，我们既往的一项回顾性研究^[8]^提示传统TBNA诊断肺癌的敏感性为61.11%，而本研究中EBUS-TBNA诊断肺癌的敏感性为96.67%，高于传统TBNA，相似的结果见于李等^[9]^的研究中。

本研究EBUS-TBNA对肺癌诊断敏感性为96.67%，结果类似采用快速现场细胞学配合诊断的研究^[5]^。其中假阴性的2例患者中1例为LN较大，中央有坏死，仅穿刺1次获得组织后即停止穿刺，假阴性的原因考虑主要为穿刺次数不够，已有研究显示在无快速现场细胞学的配合下，在判断LN转移时，需要最少3次穿刺，除非得到足够的组织，在这样的病例中，2次穿刺亦可。采取上述原则，穿刺的敏感性可以达到91%-95%，阴性预测值为96%-97%^[10]^。另外1例患者可能与定位不准、穿刺针并未准确穿入转移的LN或LN的转移部位有关。

根据我们学习曲线的经验，在最初的30例EBUSTBNA进行检查的患者中，EBUS-TBNA对肺癌诊断敏感性为86.96%，阴性预测率为70%，结果类似国外同类包括学习曲线过程的研究^[11-14]^。其中假阴性的3例患者全部在前5例行EBUS-TBNA操作的患者中，假阴性的病例原因考虑主要为穿刺次数不够。我们认为在操作5例之后应该获得不低于90%的诊断肺癌的阳性率。有研究^[14]^报道对胸外科医生而言，在经过10例的训练之后，敏感性可以从50%上升到96%。术者尽管为肺内科医生，但具有熟练使用常规气管镜并且进行过30例左右的传统TBNA操作经验，所以可以获得较高的阳性率。

EBUS-TBNA相对安全，能耐受纤支镜检查的患者基本能耐受该项检查，并发症较低。我们的一项荟萃分析研究^[15]^表明在总计11项研究入选的1 299例患者中，仅2例慢性阻塞性肺病患者出现了并发症（0.15%），分别为气胸和术中低氧，1例进行了胸部置管引流排气，1例术后很快从低氧血症中恢复。我们的体会是术中主要并发症为穿刺点少许出血，一般无需处理或局部应用肾上腺素处理即可。较严重的潜在并发症为气胸、纵隔气肿、大血管出血等，很少出现，轻者无需处理，重者胸腔积气引流或请外科协同处理。

EBUS-TBNA微创、安全，可在局部麻醉下操作，在门诊进行，简便经济，并发症少，且对肺癌诊断符合率较高，是诊断气管旁及纵隔病变安全、有效的方法，同时对肺癌的准确分期以及治疗方案的制定有着非常重要的作用。
